# Assessment of Rice Developmental Stage Using Time Series UAV Imagery for Variable Irrigation Management

**DOI:** 10.3390/s20185354

**Published:** 2020-09-18

**Authors:** Chin-Ying Yang, Ming-Der Yang, Wei-Cheng Tseng, Yu-Chun Hsu, Guan-Sin Li, Ming-Hsin Lai, Dong-Hong Wu, Hsiu-Ying Lu

**Affiliations:** 1 Department of Agronomy, National Chung Hsing University, Taichung 40227, Taiwan; emilyyang@email.nchu.edu.tw (C.-Y.Y.); s581011@dragon.nchu.edu.tw (G.-S.L.); dhwu@tari.gov.tw (D.-H.W.); 2 Department of Civil Engineering, and Innovation and Development Center of Sustainable Agriculture, National Chung Hsing University, Taichung 40227, Taiwan; disapear1997@gmail.com (W.-C.T.); daviddrmfsltd@gmail.com (Y.-C.H.); 3 Pervasive AI Research (PAIR) Labs, Hsinchu 30010, Taiwan; 4 Crop Science Division, Taiwan Agricultural Research Institute, Taichung 41362, Taiwan; mhlai@tari.gov.tw; 5 Miaoli District Agricultural Research and Extension Station, Miaoli 36346, Taiwan; iying@mdais.gov.tw

**Keywords:** UAV, image processing, irrigation, plant height

## Abstract

Rice is one of the three major crops in the world and is the major crop in Asia. Climate change and water resource shortages may result in decreases in rice yields and possible food shortage crises. In this study, water-saving farming management was tested, and IOT field water level monitoring was used to regulate water inflow automatically. Plant height (PH) is an important phenotype to be used to determine difference in rice growth periods and yields using water-saving irrigation. An unmanned aerial vehicle (UAV) with an RGB camera captured sequential images of rice fields to estimate rice PH compared with PH measured on site for estimating rice growth stages. The test results, with two crop harvests in 2019, revealed that with adequate image calibration, the correlation coefficient between UAV-PH and field-PH was higher than 0.98, indicating that UAV images can accurately determine rice PH in the field and rice growth phase. The study demonstrated that water-saving farming is effective, decreasing water usage for the first and second crops of 2019 by 53.5% and 21.7%, respectively, without influencing the growth period and final yield. Coupled with an automated irrigation system, rice farming can be adaptive to water shortage situations.

## 1. Introduction

Global climate change is worsening, and production risk and loss of crops caused by weather disasters increase yearly. Many regions of the world face the potential problem of food shortages. In most Asian countries, rice is the staple food. Rice yields are decreasing with increased cellular respiration and carbon metabolism due to increasing global average temperature [1,2,3]. Thus, effectively increasing rice yields under adversity is a prominent challenge requiring urgent attention. Asia is the main region of rice production, with a farming area accounting for 90% of the entire world. Agricultural irrigation accounts for 80% of fresh water use in Asia, and over 90% of irrigation is for rice production [4]. Inundation irrigation is mostly adopted for rice farming, and the water demand for irrigation is large. Water usage periods are concentrated, making water shortage more likely. Moreover, rice farming faces problems of water resource shortage due to climate change and urbanization. By 2025, 15 million hectares of rice irrigation areas in Asia may be influenced by water shortage [5]. Aside from fertilization management and pest control at specific times, irrigation management in rice farming also requires massive labor and time. Simplified irrigation management often leads to water overflow and fertilizer loss. To improve irrigation management, the system of rice intensification depends on the regularity of the developmental stage of rice for irrigation with smaller amounts of water. In addition, the management of water, nutrients, and soil of the rice is improved, which increases the yield by 25–50% and reduces water usage by 25–50% [6]. International Rice Research Institute tested alternate wetting and drying field irrigation. The water levels were measured manually with human eyes, and the irrigation stopped when water levels were 4 cm higher than the soil surface. After water levels went to 0 cm, irrigation resumed. The test results indicated that alternate wetting and drying could reduce irrigation water usage and increase the water usage efficiency [7].

The growing period of rice is influenced by external environmental factors, such as temperature, sunshine, and rainfall, and yield is finally influenced [8]. Field water and nutrient management is key to increasing rice yield [9,10]. Plant height (PH), leaf area index, chlorophyll, plant nitrogen content, and soil nitrogen content are essential parameters for the estimation of crop yield [11,12,13,14]. Amongst others PH is a vital phenotypic trait in crop growth that influences lodging, biomass, and yield [15]. Conventional PH measurement with vision or ruler requires substantial labor and time; therefore, it cannot be continually used for the measurement of rice PH over large areas. In addition, human error or harm to rice due to people entering the field may also occur.

To improve the quality and quantity of staple crop production and address problems arising from labor shortage, production management in farming must become more automated. The use of agricultural water should be precise and labor-saving to achieve maximum production efficacy [16]. Improvements in sensor and related techniques can gradually replace human labor. For example, light detection and ranging (LiDAR), ultrasonic sensors, and RGB cameras can be used for PH measurement. LiDAR with unmanned vehicles can be used to measure wheat PH from the ground, and dynamic changes of PH can be used to estimate the wheat blossoming dates [17]. LiDAR with a field operation platform (Phenomobile Lite) can be used to measure wheat PH [18]. Ultrasonic sensors installed on agricultural vehicles were used to estimate the height and biomass of various weeds, such as *Trifolium pratense* L., *Trifolium repens* L., and *Medicago sativa* L. [19]. Imaging technology continues to progress, and RGB cameras are extensively used due to their favorable spatial resolution and low cost [20]. Sensors with RGB cameras can be used for the estimation of biomass [11,21,22,23,24,25] and breeding phenotypic trait screening [26,27,28,29]. RGB and multispectral cameras were evaluated for monitoring flowering intensity of four cool-season crops to enhance the accuracy and efficiency in quantifying flowering traits [30]. Unmanned aerial vehicles (UAVs) with RGB cameras are used with crops such as sorghum, corn, tomato, and rice. Information on the agricultural environment, crop surveillance, crop phenotypic traits, crop symptoms, and disaster damage research can be conducted or gathered [29,31,32,33,34,35]. However, UAVs are currently less used for long-term continual observation.

In this study, alternative wet and dry (AWD) water-saving rice farming was compared with conventional planting (CP). An automated control irrigation system with water level sensors was set up in fields, and a commercial UAV was used to capture large-scale and continual field images. 3D models were manufactured, and the rice PH was analyzed. Image interpretation was used along with actual field phenotypic traits survey, yield research, and weather observation for the rice growth period. A large-area, long-term, continual, and nondestructive observation of rice growth was achieved. The use of labor resources was reduced, as was investigation time. This method also assisted managers in making field management decisions to respond to environmental changes and to undertake water-saving rice farming management.

## 2. Experiment and Analysis Methods

### 2.1. Experiment Location and Materials

**Figure 1:**
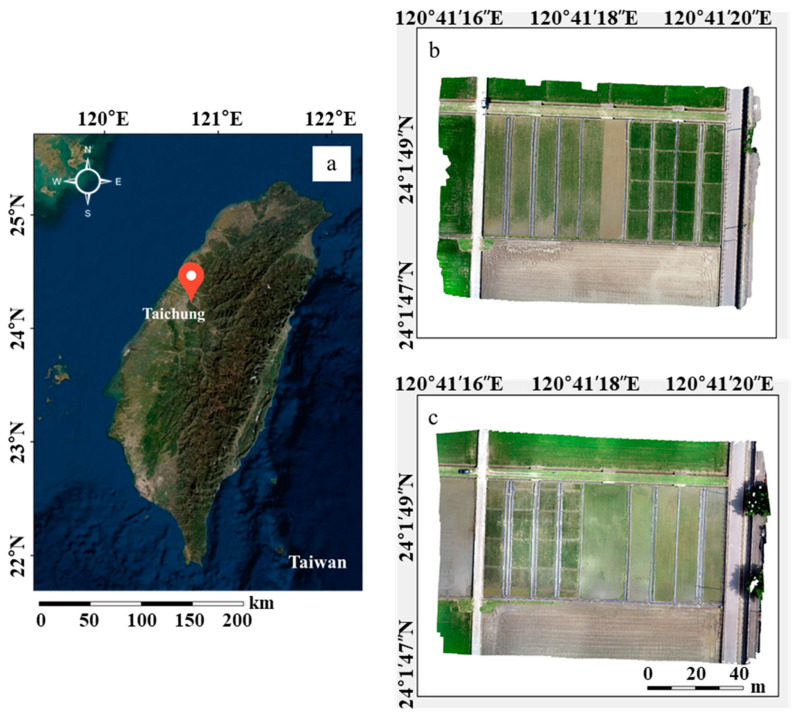
The location of the study area and rice crop in different season. (**a**) Study area located in Taichung, Taiwan. The orthomosaic image produced from the unmanned aerial vehicle (UAV) flight in 2019; (**b**) first crop season; and (**c**) second crop season.

The experiment was conducted at the experimental field of Taiwan Agriculture Research Institute (TARI) in Taichung City, Taiwan (24.10° N, 120.41° E; Figure 1a). The climate is subtropical, with an average annual temperature of 24.4 °C and annual rainfall of 2507 mm. The experimental field is an independent experimental area managed by professional personnel with sufficient resources for the experiment. The experimental rice variety was Tainung 71 (TNG71), which has decent plant type, favorable cold tolerance, shorter growth period, stable yield, and long resistance to most pests and diseases. It is extensively grown in Taiwan. Two rice crops, first and second, are grown in one year in Taiwan. The two crops have been grown under different climate conditions. The farming period of the first crop is longer than the second, and the yield of the first crop is higher. Due to the differences between the crops, the experiment was conducted with two crops in one year. The first crop was cultivated from March to June, and the temperature changed from low to high. The second crop grew from July to October, and the temperature shift was from high to low.

### 2.2. Experimental Field Planning and Water Management

**Figure 2:**
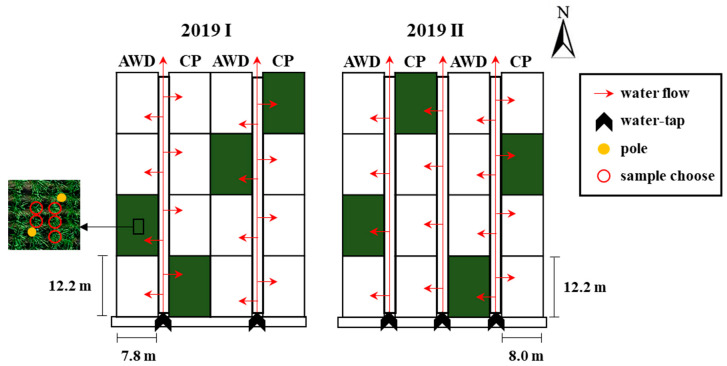
Schematic diagram of water treatment in the test field, Taiwan Agricultural Research Institute, Council of Agriculture. Green areas are experimental rice paddies in this study.

The seedling transplant of the first crop was on 8 March 2019 (Figure 1b right). The left side of Figure 1b is an image of the blank experimental area with no treatment to avoid fertilizer residue. The seedling transplant of the second crop was on 26 July 2019 (Figure 1c left). The right side of Figure 1c is the blank experimental area. The area of each field section was 97.60 m^2^ (8.0 m × 12.2 m), and the plant spacing was 30 cm × 21 cm. The fertilizers were basal fertilizer (#39 Biotech Organic Fertilizer 261.8 kg/ha and nitrogen fertilizer 31.4 kg/ha), top dressing (ammonium sulfate 136.1 kg/ha and nitrogen fertilizer 28.3 kg/ha), and ear dressing (ammonium sulfate 149.7 kg/ha and nitrogen fertilizer 31.4 kg/ha). Each field section had an independent water system. Irrigation water inflow was from the south, and the water outflow was to the north (Figure 2).

Two field water management methods were compared. CP followed the conventional field method, and a particular water level was maintained for the entire growth period. AWD was alternating irrigation, with the CP water level as the highest level. After the water reached the highest level, irrigation stopped until the water level decreased to 0 cm. The following day, the water level was increased to the highest level. This process was repeated until the ripening stage. The two methods were implemented in the two repeated field sections, and irrigation for both was stopped at the soil drying stage. A micro weather station system (Evolution Sensors Platform, ESP-4WPB001) was used in the experimental field to monitor weather data, such as temperature and rainfall. A smart water level sensor (SW01T06001R) was placed in the center of each field to monitor real-time water levels. In addition, automated remote control (SW10C09007DN50) was used to open and close the water gate, and an electromagnetic flowmeter was used to document water usage. The ESP weather station and sensors provided the functions of water level control, field water use monitoring, and agricultural environment monitoring.

### 2.3. PH Measurement

**Figure 3:**
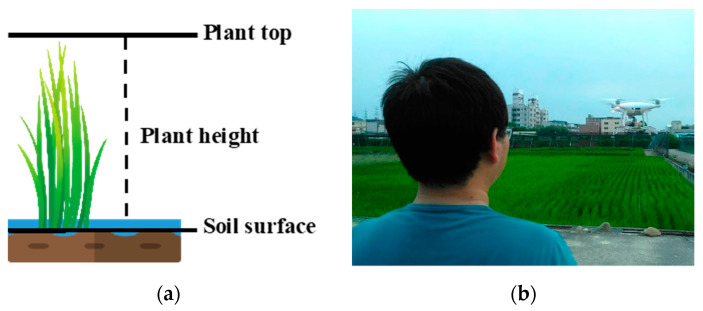
Overview of field plant height survey. (**a**) Plant height measured from soil surface to plant top using rulers; (**b**) Plant height generated by UAV.

After the rice seedlings were transplanted to the field, PH was measured weekly after 11 days of the survival period. Areas with water inflow or outflow, human destruction, or damage from external force were avoided. Two poles were set up in every field section, and five plants were selected in the area to measure the distance between the ground and the highest point of the plant with a wooden ruler (minimum scale of 0.1 cm). The average value was obtained as the field PH (Figure 3a). PH measurement stopped when the PH remained constant, in the heading stage.

### 2.4. UAV Image Shooting

The general commercial UAV DJI Phantom 4 Pro was employed (Figure 3b) with an RGB camera of 20 million pixels (5472 × 3648) and 8.8 mm focal length. The camera has a field of view of 84°, and the images were captured with shutter priority and a constant exposure value to avoid blurry images caused by slow shutter speed. The image size was 3:2 to avoid edge deformation caused by wide angles. To obtain superior rice plant details, the flying height was 20 m and the ground sample distance was 5.0 mm/pixel. The high overlapping image capture was adopted to establish favorable rice 3D models, with 85% forward overlap and 85% side overlap. Image quality is the key to image processing [36]. Four control points were set up around the shooting area as the overlapping basis for image coordinates in multiple stages. The shooting duration and weather at the time were documented. The images were checked for problems, such as blur, overexposure, and damage. The UAV image shooting duration and the on site investigation time were basically identical but were adjusted according to the weather. Aerial monitoring was conducted once a week until on site investigation determined no change in PH.

### 2.5. Image Analysis

**Figure 4:**
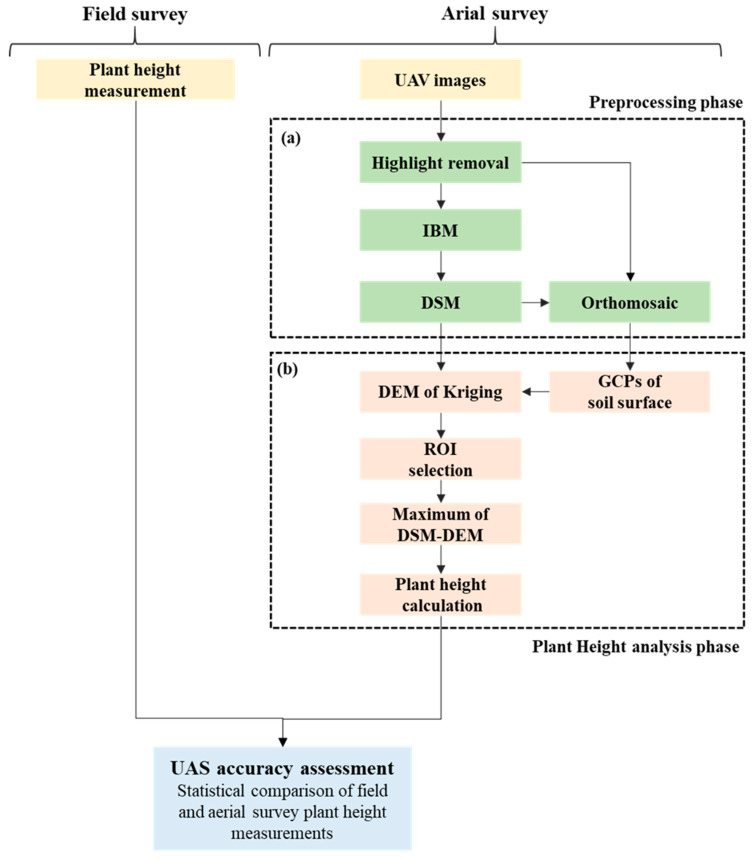
Workflow of plant height generated by UAV images including (**a**) DSM generation and (**b**) plant height model generation.

The image analysis process, including geometric calibration and radiation calibration, is depicted in Figure 4. First, the highlight (HL) pixels were removed, and image-based modeling was used to make digital surface models (DSMs) and orthomosaic images. A kriging method was used to make digital elevation models (DEMs) of the field surface in many studies [28,29,37,38], especially for a non-linear interpolation with a limited control points [39]. The pixels of the region of interest were selected on the four corners of each paddy to generate a paddy DEM, and a plant height model (PHM) was established to calculate PH by deducting the DEM results from the DSM results.

#### 2.5.1. HL Pixel Removal

**Figure 5:**
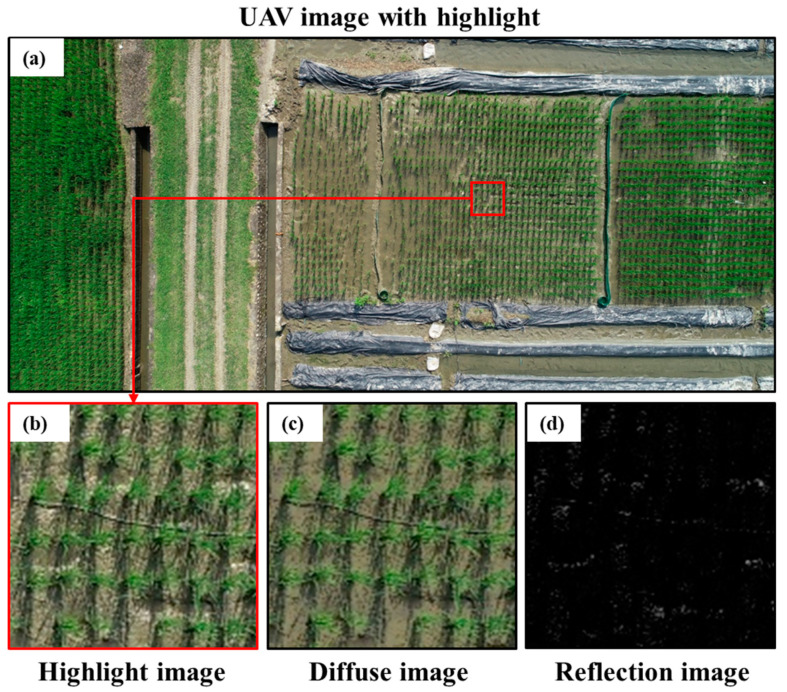
(**a**) Original UAV image; (**b**) highlight image; (**c**) diffuse image; and (**d**) reflection image.

The water surface of a rice field reflects sunlight and causes HL problems with overexposure. This frequently occurred in the seedling and early tillering stages (Figure 5a). HL removal methods include methods by Tan and Ikeuchi, Yoon et al., Shen et al., Shen and Cai, Yang et al., Shen and Zheng, and Akashi and Okatan [40,41,42,43,44,45,46]. The method of Shen and Cai [42] is the fastest so it was selected for this study, considering the large number of UAV pictures and their high resolution.

Light intensity and the gray scale value have a linear relationship in HL, and HL is formed by the overlapping of diffusion and reflection. The diffuse images (Figure 5c) and reflection images (Figure 5d) were separate from HL images (Figure 5b). Python, Intel Core i7-4790 CPU, and 32 GB memory were used for the calculation. The HL processing of each 20-million-pixel UAV image required six seconds. Each UAV mission collected 500 to 600 images, and the HL processing time was approximately 1 h.

#### 2.5.2. Image Modeling

Compared with traditional aerial surveying, image-based modeling does not depend on high-accuracy equipment to document exterior orientation parameters [47]. Image characteristics are automatically searched using Scale-Invariant Feature Transform with three or more images with an overlapping rate of over 67%. Structure from Motion was used to calculate the interior and exterior orientation parameters of the cameras and scattered point clouds. Condense point clouds, DSM, and orthomosaic images were made with Multi-View Stereo, substantially reducing the modeling cost [23,47,48]. Agisoft Metashape (1.16.1) was used for the procedure. Rice plants are not steadily fixed and are easily influenced by wind. For feature point detection, the original image resolution was decreased to one-quarter to reduce the effect of leave movement or the tiny leave tips, and the number of stable feature points was increased to establish stable interior and exterior orientation parameters. Original resolution images were used to make condense point clouds to obtain more feature match points. In addition, the filter function was turned off to avoid the removal of point clouds at the upper part of the plants. Finally, the control points were used to calibrate DSM and orthomosaic images. In addition, the images were positioned geometrically using the TWD1997 coordinate system for image overlapping and comparison of multiple periods.

#### 2.5.3. Kriging Spatial Interpolation

Considering the varying heights of the surfaces in different field sections as the basis of PH calculation, kriging spatial interpolation in ArcMap (10.2, Esri Inc., Redlands, CA, USA) was adopted [49,50]. Exposed soil points with no plant coverage in the four corners of the orthomosaic images were identified as the known points, and the kriging interpolation was used to obtain the DEM of each field section. In the kriging assumption, height is related to distance [50].

(1) $$\;\hat{Z}\left(s_{0}\right)=\overset{N}{\underset{i=1}{\sum }}\lambda_{i}Z\left(s_{i}\right)$$

$Z\left(s_{0}\right)$ is the height prediction value, and $s_{0}$ is the pixel coordinate. $Z\left(s_{i}\right)$ is the height of the *i*-th point, and $s_{i}$ is the pixel coordinate of the *i*-th point. $\lambda_{i}$ is the weight of the *i*-th point, and represents the correlation between the known point and the predicted point. The semivariogram was used to describe the spatial correlation between the points.

(2) $$\sigma_{i}=\frac{\sum_{j=1}^{k}{\left(s_{i}-s_{j}\right)}^{2}}{2K}$$

In which, $\sigma_{i}$ is the semivariogram of the *i*-th point, $s_{i}$ is the coordinate of the *i*-th point, and $s_{j}$ is the coordinate of the K points other than the *i*-th point. Every known point has a semivariogram and its predicted value. In this study, a spherical model was adopted for semivariogram. They can compose a distribution graph and a fitting function, and $\lambda_{i}$ is given to predict the height of unknown points.

**Figure 6:**
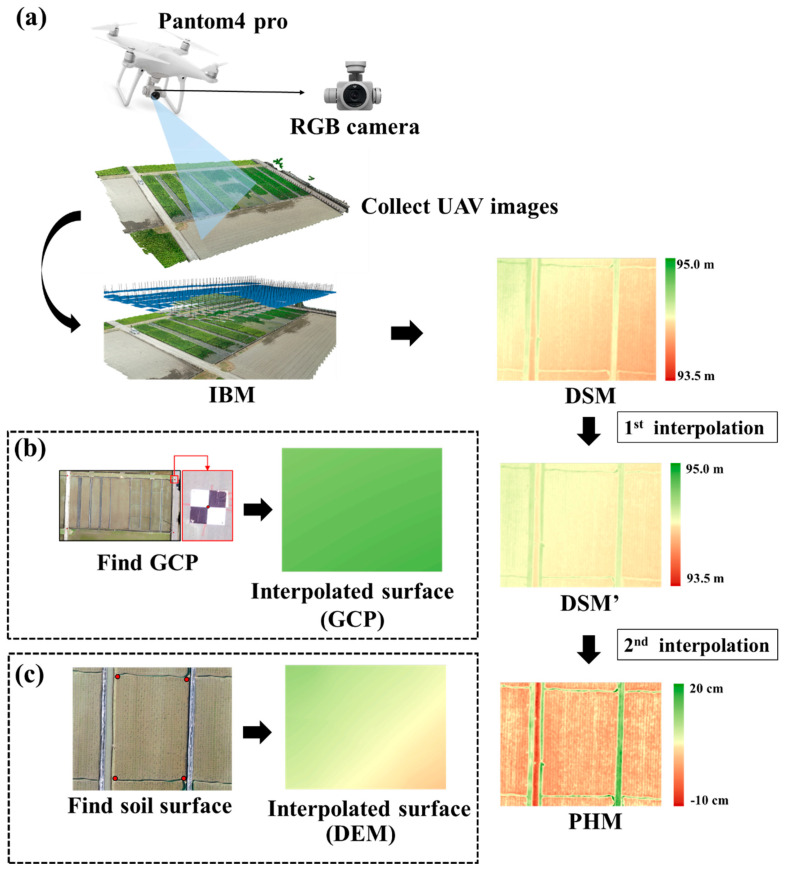
An overview of kriging for image interpolation. (**a**) DSM generated by UAV images; (**b**) modified DSM by kriging interpolation based on ground control points; and (**c**) generated DEM by kriging interpolation based on bare ground points.

To reduce DSM height errors in UAV images (Figure 6a) and generate DEMs of the soil surface, kriging interpolation was conducted to calibrate DSMs, using the control points to reduce height error (Figure 6b) and generate soil surface DEM, using the four known points in the field section (Figure 6c).

#### 2.5.4. PH Calculation and Analysis

PHM was generated by subtracting the DEM from the DSM [37,49]. The average value of the selected highest 100 points of PHM represented UAV-PH. In addition, a regression analysis was conducted with UAV-PH and field-PH. The coefficient of determination (R^2^) was used to evaluate the regression performance.

(3) $$R^{2}=1-\frac{\sum {\left(y_{i}-\bar{y}\right)}^{2}}{\sum {\left(f_{i}-\bar{y}\right)}^{2}}$$

In the formula, $y_{i}$ is the field-PH, $\bar{y}$ is the mean field-PH, and $f_{i}$ is the UAV-PH.

## 3. Results and Discussion

UAV images of the field were collected every week through on site investigation and manual measurement of PH (a total of 22 times). The flight altitude was 20 ± 2 m, and GSD was 4.58–5.48 mm/pixel. Weather changes, such as temperature, wind speed, and humidity had to be considered for UAV flight, and flight dates were advanced or postponed 1 to 3 days according to weather forecasts (Table 1).

**Figure 7:**
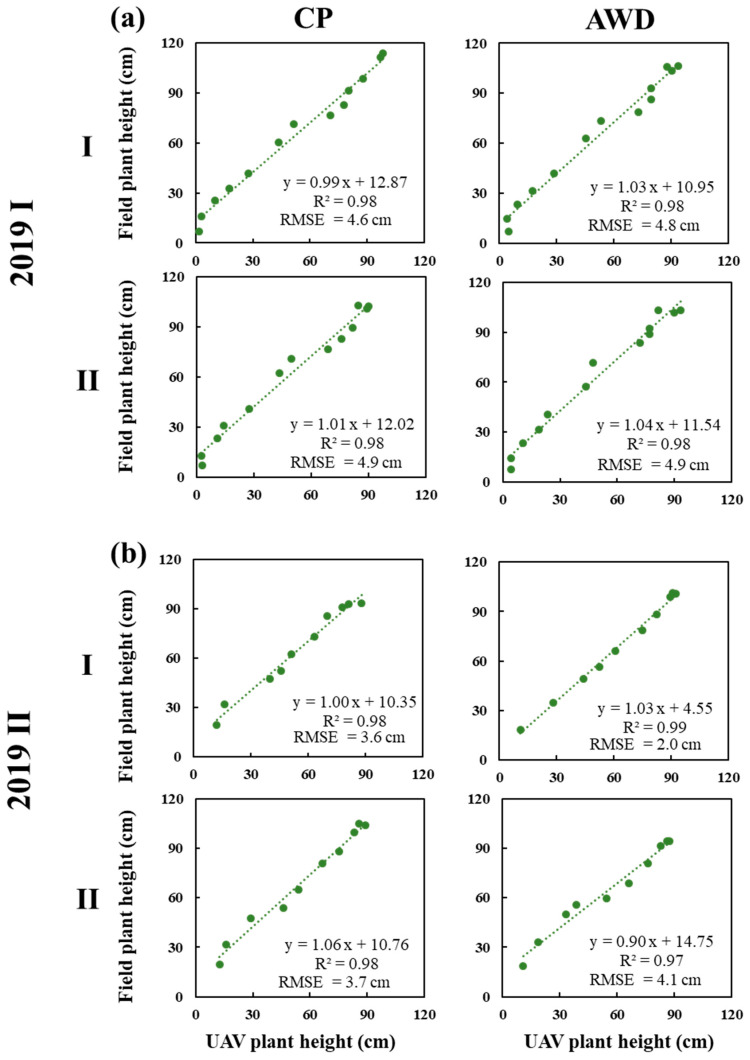
Comparison between the mean plant height derived from UAV measurements and plant height measured manually of TNG71 rice variety in the field under different water treatment (conventional planting (CP) and alternative wet and dry (AWD)) in (**a**) the first crop season; and (**b**) the second crop season.

In literature, the UAV-PH studies were applied to dryland crops, such as upland rice (without irrigation) [51], wheat [52], and maize [28]. Rice is an irrigated and inundated crop. The paddy needs to maintain a water level at a certain height, and the UAV imagery is prone to get highlight spots due to sun reflection from water surface, which are uncontrollable environmental variables and seriously deteriorate orthomosaic imagery quality. Thus, highlight problem is a big issue and needs to be dealt with before image processing. This study proposes a UAV image quality improving procedure, which can be applied to both irrigated and dryland crop. Relying on HL-removed UAV images, DSM and DEM were generated and their difference calculated for PHM; UAV-PH was produced as an area-basis and compared to point-based field-PH. The coefficient of determination (R^2^) of UAV-PH and field-PH was 0.98 for both CP and AWD in the first crop season (Figure 7a) and improved to 0.99 for both CP and AWD in the second crop season (Figure 7b). HL removal was proven effective in reducing the effect of specular reflection and increasing the accuracy of UAV-PH. A high correlation, with an R^2^ close 1, between UAV-PH and field-PH, without significant difference with water treatment (CP or AWD), was observed (Figure 7).

**Figure 8:**
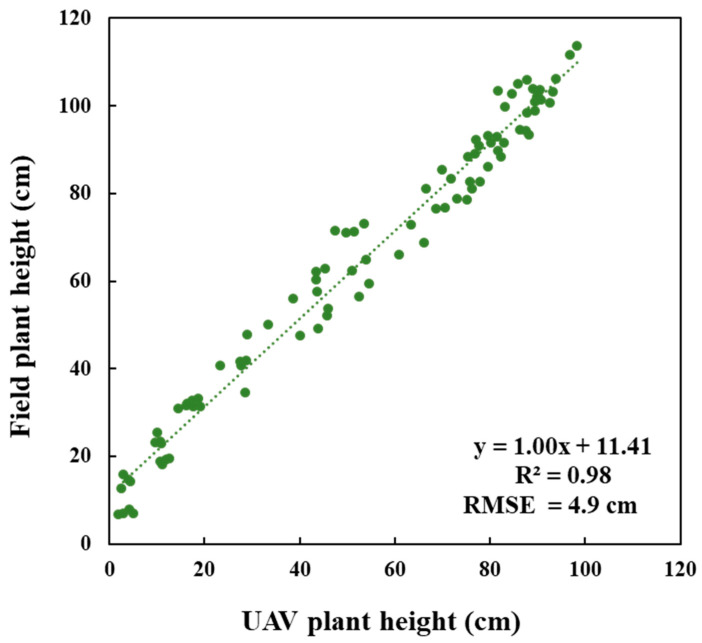
Correlation between UAV plant height and field plant height.

Over two crop seasons, with three developmental stages and two water treatments, PH was generated from UAV images with high reliability. For all collected data (80 heterogeneous samples), UAV-PH exhibited a 1:1 linear relationship with field-PH, with a promising estimation capability, with an R^2^ of 0.9753 (Figure 8). Essentially the UAV-derived PH systematically underestimates the ground truthing PH with approximately 11cm, because the image-based modeling technique lacks the ability to accurately reconstruct the tiny leave tips. This result is consistent with previous findings in [51,53].

**Figure 9:**
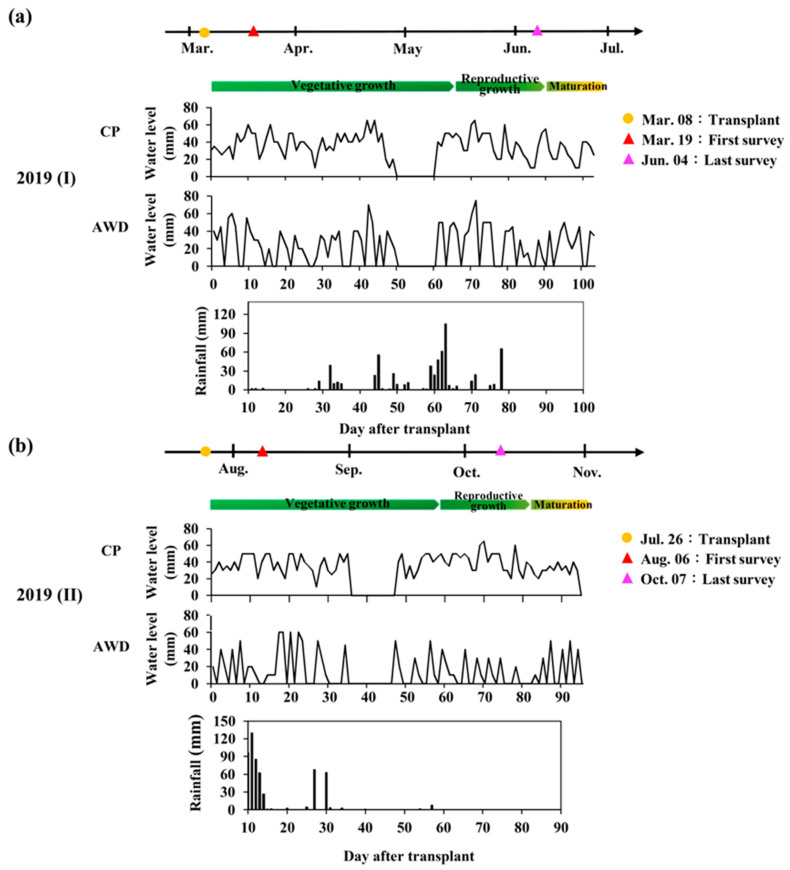
The cultivation calendar of rice cultivar TNG71 and the variation of water level and rainfall during days after transplant under different water treatments in (**a**) the first crop season; and (**b**) the second crop season.

**Figure 10:**
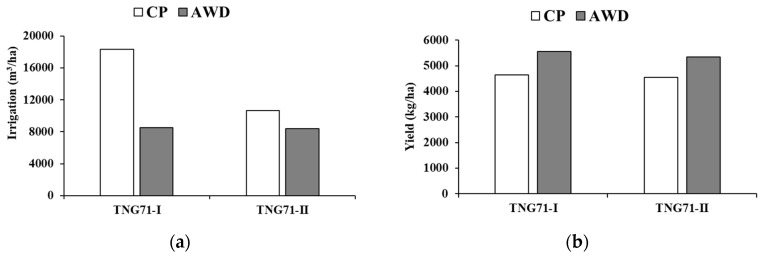
(**a**) Field irrigation; (**b**) yield of TNG71 under different water treatments in the first and second crop seasons.

With CP, the water level of the field was maintained at 5 cm, and irrigation was stopped at the soil drying stage. The water management of AWD involved waiting until the water level dropped to 0 cm, and the irrigation resumed on the next day until the water level was again 5 cm. The process was repeated until the harvest stage, although irrigation stopped for soil drying (Figure 9). The irrigation amounts of the first and second crops under CP management were 18,297.1 m^3^/ha and 10,672.8 m^3^/ha, respectively. The irrigation amount of the first and second crops under AWD management were 8516.7 m^3^/ha and 8358.4 m^3^/ha, respectively. The water-saving rate of rice farming using AWD was 53.5% for the first crop and 21.7% for the second (Figure 10). Due to the plum monsoon during the first crop, effective management saved even more water resources.

**Figure 11:**
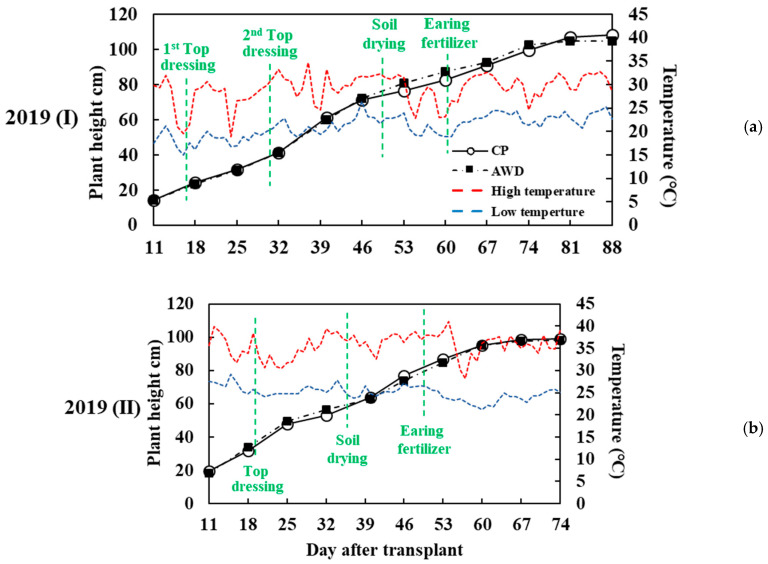
Plant height and the variation of the highest and lowest temperature with cultivation processes in (**a**) the first crop season; and (**b**) the second crop season.

Figure 11 depicts the cultivation schedules of the first and second crops. The first top dressing was conducted on the 17th day from transplant date in the first crop, and the second top dressing was conducted on the 31st day. Field drying began on the 49th day, and ear dressing was performed on the 61st day. For the second crop, the top dressing was on the 19th day from transplant date. Soil drying was begun on the 35th day, and the ear dressing was performed on the 48th day. Effective accumulative temperature can be accumulated as growing degree days (GDD), a weather-based indicator for assessing crop development [54]:

(4) $$\mathrm{GDD}=\sum \left\{\frac{T_{\mathrm{Max}}+T_{\min}}{2}-T_{b}\right\}$$

**Figure 12:**
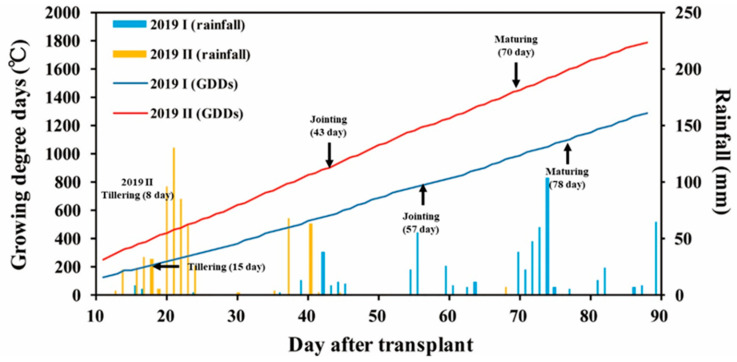
The growing degree days of TNG711 until panicle initiation and rainfall in the first and second crop seasons.

T_Max_ is the highest temperature of the day, and T_min_ is the lowest temperature of the day. T_b_ is the base temperature for growth, and it is assumed to be 10 °C generally for rice growth [55]. In the past, GDD was used to estimate the growth period of the crop. However, the global climate change is accelerating. The rising temperature may influence the growth period of crops, and the accuracy of GDD may be affected. Figure 12 presents the GDD of the first and second crops in 2019. The GDD of the second crop was higher than that of the first crop. Consequently, its total required growth period was approximately two weeks shorter than that of the first crop. The growth degree model of the second crop in 2019 until the panicle initiation was compared with the experience-based estimation value, and the difference was large (311 °C higher, 38.2%; Table 2). This indicates that the effective accumulation temperature was reduced under higher temperature. If the conventional estimation formula is used, this may result in a substantial increase in the green grain ratio at harvest. In addition, GDD is influenced by local climate and growth conditions. Consequently, using GDD to estimate the growth period of crops requires the premise of a stable climate, and its applicability will gradually decrease under global warming.

Rice developmental stages can be divided into vegetative growth, reproductive growth, and maturation. At the stage of vegetative growth, including seedling and tillering, roots, stems, and leaves are mainly grown. Thus, the plants absorb water and nutrients, and photosynthesis efficiency is increased, providing nutrients to roots, stems, and leaves. In seedling, leaf number increases, and PH increases slowly. Tillering is subdivided into early tillering, middle tillering, and maximum tillering, during which PH increases rapidly until the tiller number stops growing. The stage of reproductive growth includes panicle initiation, booting, heading, and full heading. The maturation stage includes the milk, soft dough, and hard dough stages and maturity. The reproductive growth stage starts from the differentiation of panicle initiation and continues to heading and flowering. In this period, rice PH grows slowly. When a rice plant ripens, its PH stops growing.

Experts investigated on site growth every week to match it with the UAV-PH. If the transformation of a growth period occurred, a dividing point was set between investigations. According to the on site investigations (Table 3), the seedling stage of the first crop was days 1–14, and the tillering stage was days 15–56. The vegetation stage was days 57–77, and the ripening stage was day 78–103. The seedling stage of the second crop of 2019 was days 1–7, and the tillering stage was days 8–42. The vegetation stage was days 43–70, and the ripening stage was days 71–95.

**Figure 13:**
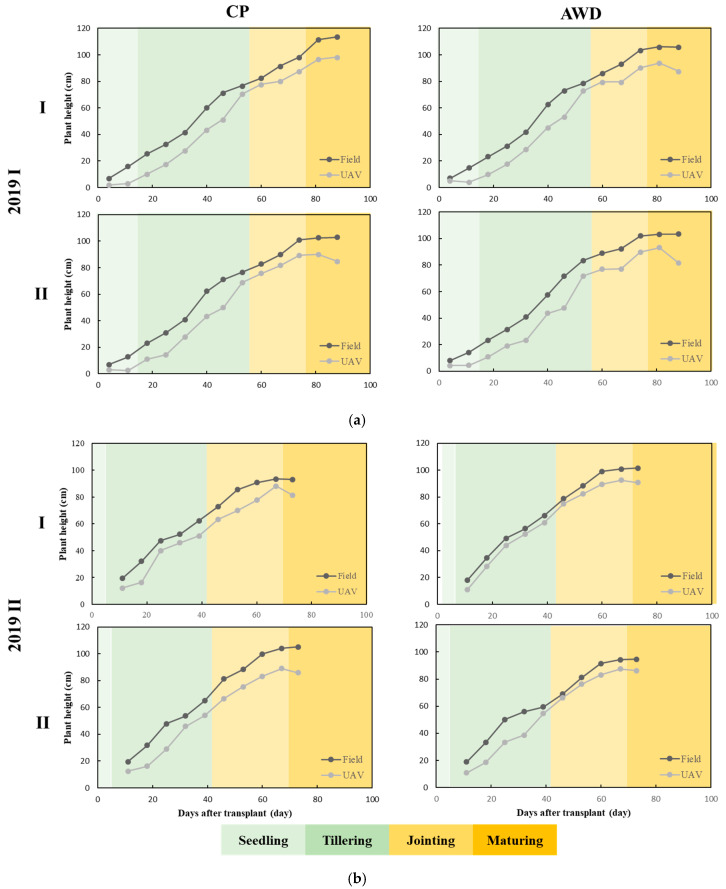
Comparison between UAV plant height and field plant height with developmental stages in (**a**) the first crop season; and (**b**) the second crop season.

The determination of the rice growth period is typically made through expert on site investigation and experiment. This requires considerable labor, cost, and time. In addition, destructive sampling is adopted, which influences rice growth in the field. In this study, UAV-PH was used to estimate the growth period. The field did not need to be entered, so influence on the crop was limited. Little labor was used for large-scale investigation. In rice growth, periods with apparent PH changes are vegetative growth and reproductive growth. Growth speed is related to temperature and differs between the first and second crop due to the different seasons. The correspondence between field-PH and UAV-PH is depicted in Figure 13. The vegetative growth, reproductive growth, and ripening stages can be clearly distinguished. The PH curve slope in Figure 13 indicates that for the first crop, PH had a high growth rate from day 11 to day 32 after transplant, the seedling stage to the tillering middle stage; it was slower relative to the second crop. After the two top dressings, PH change was faster, and it was fastest at the tillering stage. The purpose of soil drying is to expand the range and depth of the rice root system. The rice roots become stronger, and lodging resistance is increased. In addition, tillering can be inhibited by insufficient water uptake. The useless plant nutrient loss can be reduced, and the richness of the rice ears is increased. Consequently, soil drying timing is a key decision in rice farming management.

**Figure 14:**
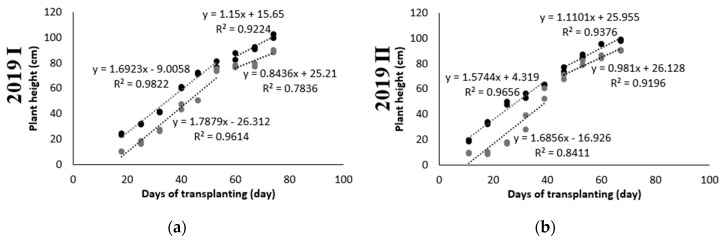
Rice growth rates during three developmental stages in (**a**) 2019 I; and (**b**) 2019 II.

According to the experiment results, PH at soil drying for the first crop was approximately 75 cm, and the PH of the second was approximately 59 cm. At soil drying, rice PH change is slow. After drying, ear dressing is implemented, and plant stems elongate. PH then increases gradually. When plant stems stop growing, PH ends and the booting stage is entered. PH changes in the initial stages of the second crop are slightly different from those of the first. The growing environment of the second crop was from high to low temperature, and the cumulative temperature change rate was large. Consequently, in the initial stages, PH growth rate was high. Growth was also faster at the tillering stage. After ear dressing, PH change was identical to that of the first crop, and the rate of change stopped increasing. In the ripening stage, PH stopped increasing. Average PHs at the ripening stage of the first and second crops were 107 cm and 99 cm, respectively. PH at the ripening stage of the first crop was approximately 6–8 cm higher than that of the second. In the later ripening stage, PH slightly decreased due to the rice ear bending. In conclusion, the PH changes of rice can be divided into the three stages of vegetative growth, reproductive growth, and ripening. The PH changes at the three stages are steep, shallow, and flat, respectively (Figure 14).

**Figure 15:**
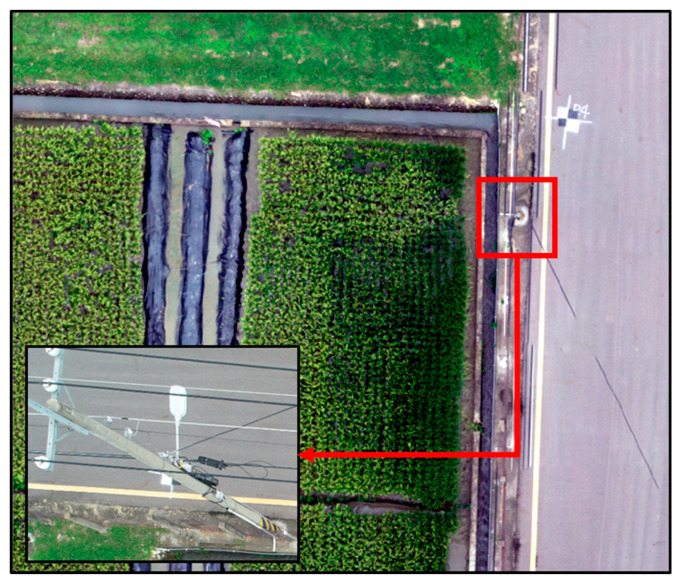
Street light causing over-growing PH and preventing from blooming in CP-I in the first crop season.

**Figure 16:**
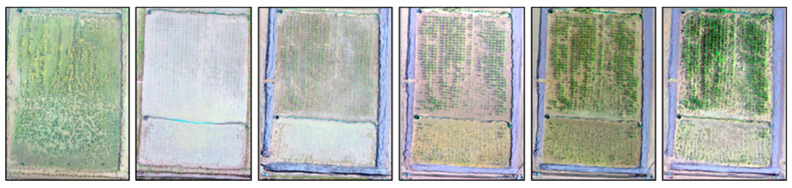
Low soil levels causing rice death in AWD-I in the second crop season.

**Figure 17:**
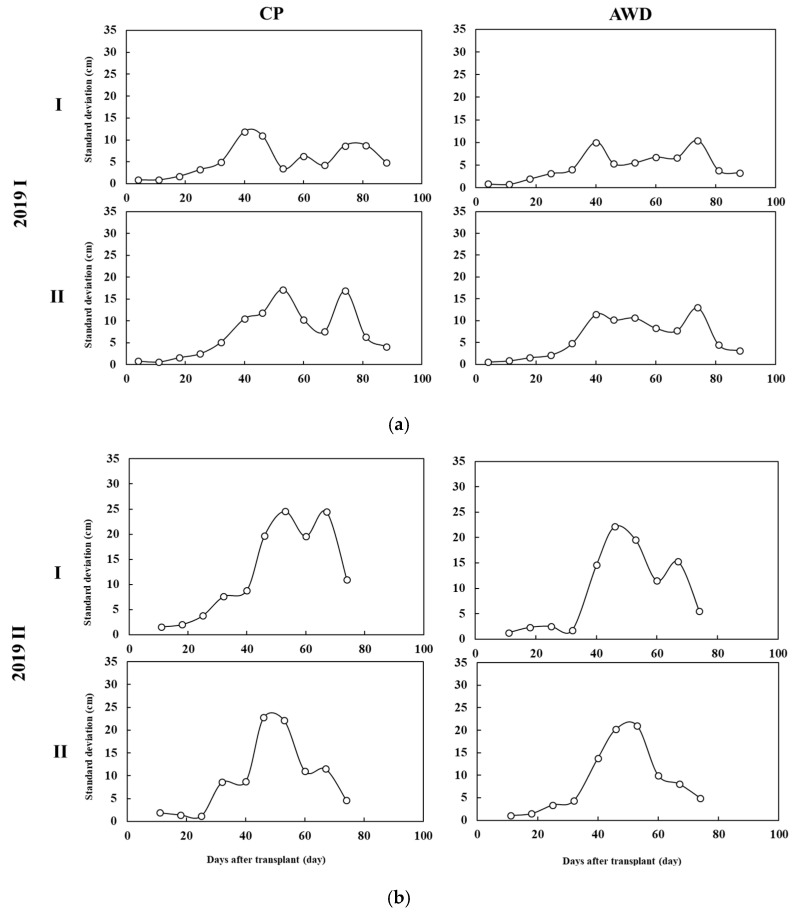
Standard deviation of UAV plant height in (**a**) the first and (**b**) the second crop seasons under different water treatments (CP and AWD).

UAV images can be also used to identify management problems in field farming. For example, rice in the CP-I section in the first crop was influenced heavily by street lamps. Rice is a short-daylight plant, and long-term lighting from street lamps delays flowering, which resulted in abnormally high PH in that section (Figure 15). In the second crop, the CP-I section was the transplanter turning area. The soil was overly compressed, and the surface was lower. Thus, ponding resulted in rice dying (Figure 16). Causes from different water management should be excluded, however. A high standard deviation of PHM (Figure 17) suggests high diversity of rice cultivation in the second crop season, which reflects either loose management or a large climate impact. Therefore, the second crop season produced a comparatively low yield and poor quality of grains. Overall, area-based UAV-PH provides more detailed growth information than point-based field-PH as a reference for rice cultivation actions.

Although UAV-PH and field-PH exhibited extremely high correlations, their analyses were influenced by numerous factors. These include the HL problem in the seedling stage, lower PH of seedlings resulting in the human facility (such as the water level meter) influencing plant reflection, and too many leaves after the tillering stage shading the soil and influencing the DEM estimation. In addition, although PH directly reflects the growing situation, it is related to rice variety. Currently, only phenotypic trait information of TNG 71 of one year has been collected. In the future, the method of this study can be used to collect field information on a large scale. PH can be an effective indicator for the determination and interpretation of growth stages of different types of rice.

## 4. Conclusions

Due to increasing water scarcity, the application of variable rate irrigation to rice cultivation is becoming more popular. In this study, the two irrigation methods of CP and AWD were investigated, and a UAV was used to obtain instantaneous field information. A fast and accurate crop observation method was established as a tool for PH trait investigation of field crops. The method may provide a reference for water-saving farming management applications in the field.

Compared with CP, the AWD rice irrigation method used in this study resulted in 53.5% and 21.7% water use reductions in the first and second crops, respectively. In addition, the phenotypic traits and growth of the rice PH was not influenced, demonstrating the water-saving effectiveness of AWD. A PH evaluation system was also established in this study. First, HL of the field was removed. Kriging was used to generate DEMs and PHMs with the ground control points. The obtained UAV-PH had favorable outcomes in the first and second crops of both AWD and CP (R^2^ = 0.98, 0.99). The continual change of UAV-PH can respond to the key growth stages of rice (vegetative growth stage, reproductive growth stage, and ripening stage). It provides a favorable quantification reference for field irrigation management, fertilization timing, and soil drying decision-making. In addition, the area difference analysis using UAV-PH can identify field management problems such as abnormal death of seedlings and the influence of the surrounding environment. Thus, timely correction of a framing method can be undertaken. This study established a method for long-term continual rice PH trend identification on a large scale that can be effective for the estimation of rice growth stages. Accordingly, appropriate irrigation, fertilization, and soil drying treatment can be conducted.

In the future, UAVs can be used for long-term, continual monitoring of rice phenotypic traits over large areas. Automated UAV imaging connected with cloud image processing will allow the detection of rice growth over large areas with little labor, and adequate field farming management information can be provided. With an automated irrigation system, the water shortage adaptability of rice farming can be effectively improved. In addition, the rice harvest stage can be predicted, and dynamic rice yield can be estimated. Finally, food safety management evaluation information can be provided to administrators to ensure food safety under extreme weather.

## Figures and Tables

**Table 1:** List of UAV flights in two crop seasons.

UAV Survey Date	Field Survey Date	Altitude (m)	Ground Resolution (mm/pixel)	Coverage Area (ha)
19 March	19 March	20.3	5.1	1.2
26 March	26 March	20.2	5	1.2
2 April	2 April	21.3	5.3	1.3
9 April	9 April	21	5.2	1.3
17 April	17 April	18.1	4.5	1.2
23 April	23 April	22.2	5.5	1.3
30 April	30 April	20	5	1.2
10 May	7 May	20.9	5.2	1.2
14 May	14 May	20.6	5	1.2
21 May	21 May	21.1	5.1	1.4
29 May	28 May	20.9	5.1	1.3
4 June	4 June	19.5	5.1	1.2
6 August	6 August	20.3	5.1	1.3
12 August	13 August	18.6	4.6	1.2
20 August	20 August	19.1	4.8	1.2
28 August	27 August	21	5.3	1.3
3 September	3 September	18.3	4.6	1.2
10 September	10 September	20.4	5.1	1.3
17 September	17 September	21.2	5.3	1.3
24 September	24 September	22.1	5.5	1.3
1 October	1 October	22.1	5.5	1.3
8 October	7 October	21.4	5.3	1.3

**Table 2:** Comparison of the growing degree days of TNG71 predicted by a previous model and the field survey in the first and second crop seasons in 2019.

Variety	Developmental Stage	Crop	GDDs (°C) ^1^	Reference
TNG71	Transplanting to panicle initiation	I, II	814.2	[56]
		I	829.85	Our data
		II	1125.05	

^1^ GDDs: growing degree days.

**Table 3:** The growing degree days (GDDs) and plant height of TNG71 rice variety planted under different water treatment.

Year	Crop Season	Date	Days	GDDs (°C)	UAV Plant Height (cm)		Developmental Stage
					CP	AWD	
2019	I	19 March	11	126.2	2.7	4.1	Seeding
		26 March	18	209.6	10.5	10.2	
		2 April	25	299.9	15.9	18.3	
		9 April	32	401.8	27.7	26	Tillering
		17 April	40	521.2	43.4	44.5	
		23 April	46	618.8	50.6	50.5	
		30 April	53	740.2	69.6	72.4	Max tiller
		7 May	60	829.8	76.8	78.2	Panicle initiation
		14 May	67	941.7	80.9	78.3	
		21 May	74	1056.8	88.6	90.1	Booting
		28 May	81	1171.4	93.4	93.6	
		4 June	88	1286.1	91.5	84.7	Flowering
	II	6 August	11	254	12.3	10.9	Seeding
		13 August	18	402	16.2	23.5	
		20 August	28	533.2	34.5	38.7	Tillering
		27 August	32	676.6	45.9	45.5	
		3 September	39	826.3	52.5	57.7	Max tiller
		10 September	46	971.9	65	70.7	Panicle initiation
		17 September	53	1125	72.6	79.3	
		24 September	60	1253.1	80.5	86.3	Booting
		1 October	67	1393.1	88.6	90.1	
		7 October	73	1513.5	83.6	88.5	Flowering

## References

[1] Long S.P., Marshall-Colon A., Zhu X.G. (2015). Meeting the global food demand of the future by engineering crop photosynthesis and yield potential. Cell.

[2] Cai Y.Y., Bandara J.S., Newth D. (2016). A framework for integrated assessment of food production economics in South Asia under climate change. Environ. Modell. Softw..

[3] Shankar K.R., Nagasree K., Nirmala G., Prasad M.S., Venkateswarlu B., Rao C.S. (2014). Climate change and agricultural adaptation in South Asia. Handbook of Climate Change Adaptation.

[4] Khepar S.D., Yadav A.K., Sondhi S.K., Siag M. (2000). Water balance model for paddy fields under intermittent irrigation practices. Irrig. Sci..

[5] Tuong T.P., Bouman B.A.M. (2003). Rice production in water-scarce environments. Water Productivity in Agriculture: Limits and Opportunities for Improvement.

[6] Satyanarayana A., Thiyagarajan T.M., Uphoff N. (2007). Opportunities for water saving with higher yield from the system of rice intensification. Irrig. Sci..

[7] Lampayan R.M., Rejesus R.M., Singleton G.R., Bouman B.A.M. (2015). Adoption and economics of alternate wetting and drying water management for irrigated lowland rice. Field Crops Res..

[8] Sanchez B., Rasmussen A., Porter J.R. (2014). Temperatures and the growth and development of maize and rice: A review. Glob. Change Biol..

[9] Adamchuk V.I., Ferguson R.B., Hergert G.W. (2010). Soil heterogeneity and crop growth. Precision Crop Protection—The Challenge and Use of Heterogeneity.

[10] Goyne P.J., Hammer G.L., Meinke H., Milroy S.P., Hare J.M. (1996). Development and use of a barley crop simulation model to evaluate production management strategies in north-eastern Australia. Aust. J. Agric. Res..

[11] Bendig J., Bolten A., Bennertz S., Broscheit J., Eichfuss S., Bareth G. (2014). Estimating biomass of barley using crop surface models (CSMs) derived from UAV-based RGB imaging. Remote Sens..

[12] Reddersen B., Fricke T., Wachendorf M. (2014). A multi-sensor approach for predicting biomass of extensively managed grassland. Comput. Electron. Agric..

[13] Thenkabail P.S., Smith R.B., De Pauw E. (2000). Hyperspectral vegetation indices and their relationships with agricultural crop characteristics. Remote Sens. Environ..

[14] Tilly N., Aasen H., Bareth G. (2015). Fusion of plant height and vegetation indices for the estimation of barley biomass. Remote Sens..

[15] Fernandez M.G.S., Becraft P.W., Yin Y.H., Lubberstedt T. (2009). From dwarves to giants? Plant height manipulation for biomass yield. Trends Plant Sci..

[16] Chartzoulakis K., Bertaki M. (2015). Sustainable water management in agriculture under climate change. Agric. Agric. Sci. Procedia.

[17] Madec S., Baret F., de Solan B., Thomas S., Dutartre D., Jezequel S., Hemmerlé M., Colombeau G., Comar A. (2017). High-throughput phenotyping of plant height: Comparing unmanned aerial vehicles and ground LiDAR estimates. Front. Plant Sci..

[18] Jimenez-Berni J.A., Deery D.M., Rozas-Larraondo P., Condon A.G., Rebetzke G.J., James R.A., Bovill W.D., Furbank R.T., Sirault X.R.R. (2018). High throughput determination of plant height, ground cover, and above-ground biomass in wheat with LiDAR. Front. Plant Sci..

[19] Fricke T., Richter F., Wachendorf M. (2011). Assessment of forage mass from grassland swards by height measurement using an ultrasonic sensor. Comput. Electron. Agric..

[20] Bendig J., Bolten A., Bareth G. (2013). UAV-based imaging for multi-temporal, very high resolution crop surface models to monitor crop growth variability. Photogramm. Fernerkund. Geoinf..

[21] Bendig J., Willkomm M., Tilly N., Gnyp M.L., Bennertz S., Qiang C., Miao Y., Lenz-Wiedemann V.I.S., Bareth G. (2013). Very high resolution crop surface models (CSMs) from UAV-based stereo images for rice growth monitoring in Northeast China. Int. Arch. Photogramm. Remote Sens. Spat. Inf. Sci..

[22] Bendig J., Yu K., Aasen H., Bolten A., Bennertz S., Broscheit J., Gnyp M.L., Bareth G. (2015). Combining UAV-based plant height from crop surface models, visible, and near infrared vegetation indices for biomass monitoring in barley. Int. J. Appl. Earth Obs. Geoinf..

[23] Yang M.D., Huang K.S., Wan J., Tsai H.P., Lin L.M. (2018). Timely and quantitative damage assessment of oyster racks using UAV images. IEEE J. Sel. Top. Appl. Earth Obs. Remote Sens..

[24] Cen H.Y., Wan L., Zhu J.P., Li Y.J., Li X.R., Zhu Y.M., Weng H.Y., Wu W.K., Yin W.X., Xu C. (2019). Dynamic monitoring of biomass of rice under different nitrogen treatments using a lightweight UAV with dual image-frame snapshot cameras. Plant Methods.

[25] Devia C.A., Rojas J.P., Petro E., Martinez C., Mondragon I.F., Patino D., Rebolledo M.C., Colorado J. (2019). High-throughput biomass estimation in rice crops using UAV multispectral imagery. J. Intell. Robot. Syst..

[26] Shimojima K., Ogawa S., Naito H., Valencia M.O., Shimizu Y., Hosoi F., Uga Y., Ishitani M., Selvaraj M.G., Omasa K. (2017). Comparison between rice plant traits and color indices calculated from UAV remote sensing images. Eco-Enginerring.

[27] Han L., Yang G.J., Dai H.Y., Yang H., Xu B., Feng H.K., Li Z.H., Yang X.D. (2019). Fuzzy clustering of maize plant-height patterns using time series of UAV remote-sensing images and variety traits. Front. Plant Sci..

[28] Han L., Yang G.J., Yang H., Xu B., Li Z.H., Yang X.D. (2018). Clustering field-based maize phenotyping of plant-height growth and canopy spectral dynamics using a UAV remote-sensing approach. Front. Plant Sci..

[29] Wang X.Q., Zhang R.Y., Song W., Han L., Liu X.L., Sun X., Luo M.J., Chen K., Zhang Y.X., Yang H. (2019). Dynamic plant height QTL revealed in maize through remote sensing phenotyping using a high-throughput unmanned aerial vehicle (UAV). Sci. Rep..

[30] Zhang C.Y., Craine W.A., McGee R.J., Vandemark G.J., Davis J.B., Brown J., Hulbert S.H., Sankaran S. (2020). Image-based phenotyping of flowering intensity in cool-season crops. Sensors.

[31] Yang M.D., Huang K.S., Kuo Y.H., Tsai H.P., Lin L.M. (2017). Spatial and Spectral Hybrid Image Classification for Rice Lodging Assessment through UAV Imagery. Remote Sens..

[32] Watanabe K., Guo W., Arai K., Takanashi H., Kajiya-Kanegae H., Kobayashi M., Yano K., Tokunaga T., Fujiwara T., Tsutsumi N. (2017). High-throughput phenotyping of sorghum plant height using an unmanned aerial vehicle and its application to genomic prediction modeling. Front. Plant Sci..

[33] Enciso J., Avila C.A., Jung J.H., Elsayed-Farag S., Chang A.J., Yeom J., Landivar J., Maeda M., Chavez J.C. (2019). Validation of agronomic UAV and field measurements for tomato varieties. Comput. Electron. Agric..

[34] Siebring J., Valente J., Franceschini M.H.D., Kamp J., Kooistra L. (2019). Object-based image analysis applied to low altitude aerial imagery for potato plant trait retrieval and pathogen detection. Sensors.

[35] Yang M.D., Tseng H.H., Hsu Y.C., Tsai H.P. (2020). Semantic segmentation using deep learning with vegetation indices for rice lodging identification in multi-date UAV visible images. Remote Sens..

[36] Yang M.D., Su T.C., Pan N.F., Yang Y.F. (2011). Systematic image quality assessment for sewer inspection. Expert Syst. Appl..

[37] Yue J.B., Yang G.J., Li C.C., Li Z.H., Wang Y.J., Feng H.K., Xu B. (2017). Estimation of winter wheat above-ground biomass using unmanned aerial vehicle-based snapshot hyperspectral sensor and crop height improved models. Remote Sens..

[38] Schirrmann M., Hamdorf A., Giebel A., Gleiniger F., Pflanz M., Dammer K.-H. (2017). Regression kriging for improving crop height models fusing ultra-sonic sensing with UAV imagery. Remote Sens..

[39] Akgul M., Yurtseven H., Gulci S., Akay A.E. (2018). Evaluation of UAV-and GNSS-based DEMs for earthwork volume. Arab. J. Sci..

[40] Tan R.T., Ikeuchi K. (2008). Separating reflection components of textured surfaces using a single image. Digitally Archiving Cultural Objects.

[41] Yoon K.J., Choi Y., Kweon I.S. Fast separation of reflection components using a specularity-invariant image representation. Proceedings of the 2006 International Conference on Image Processing (ICIP).

[42] Shen H.L., Zhang H.G., Shao S.J., Xin J.H. (2008). Chromaticity-based separation of reflection components in a single image. Pattern Recognit..

[43] Shen H.L., Cai Q.Y. (2009). Simple and efficient method for specularity removal in an image. Appl. Opt..

[44] Yang Q., Wang S., Ahuja N. (2010). Real-time specular highlight removal using bilateral filtering. European Conference on Computer Vision.

[45] Shen H.L., Zheng Z.H. (2013). Real-time highlight removal using intensity ratio. Appl. Opt..

[46] Akashi Y., Okatani T. (2014). Separation of reflection components by sparse non-negative matrix factorization. Asian Conference on Computer Vision.

[47] Yang M.D., Chao C.F., Huang K.S., Lu L.Y., Chen Y.P. (2013). Image-based 3D scene reconstruction and exploration in augmented reality. Autom. Constr..

[48] Yang M.D., Su T.C., Lin H.Y. (2018). Fusion of infrared thermal image and visible image for 3D thermal model reconstruction using smartphone sensors. Sensors.

[49] Han L., Yang G.J., Feng H.K., Zhou C.Q., Yang H., Xu B., Li Z.H., Yang X.D. (2018). Quantitative identification of maize lodging-causing feature factors using unmanned aerial vehicle images and a nomogram computation. Remote Sens..

[50] Wang K.H., Chu T., Yang M.D., Chen M.C. (2020). Geostatistical based models for the spatial adjustment of radar rainfall data in typhoon events at a high-elevation river watershed. Remote Sens..

[51] Kawamura K., Asai H., Yasuda T., Khanthavong P., Soisouvanh P., Phongchanmixay S. (2020). Field phenotyping of plant height in an upland rice field in Laos using low-cost small unmanned aerial vehicles (UAVs). Plant Prod. Sci..

[52] Holman F.H., Riche A.B., Michalski A., Castle M., Wooster M.J., Hawkesford M.J. (2016). High throughput field phenotyping of wheat plant height and growth rate in field plot trials using UAV based remote sensing. Remote Sens..

[53] Bareth G., Bendig J., Tilly N., Hoffmeister D., Aasen H., Bolten A. (2016). A comparison of UAV-and TLS-derived plant height for crop monitoring: Using polygon grids for the analysis of crop surface models (CSMs). Photogramm. Fernerkund. Geoinf..

[54] McMaster G.S., Wilhelm W.W. (1997). Growing degree-days: One equation, two interpretations. Agric. For. Meteorol..

[55] Frizzell D.L., Branson J.D., Wilson C.E., Norman R.J., Moldenhauer K.A.K., Gibbons J.W. (2010). Development of degree-day 50 thermal unit thresholds for new rice cultivars. BR Wells Rice Res. Ser. Ark. Agric. Exp. Stn. Univ. Ark..

[56] Lin S.H., Lu C.T., Jwo W.S., Lu H.Y. (2014). Establishment and validation of prediction model for rice growth stages. J. Taiwan Agric. Res..

